# Cataract-causing mutation S228P promotes βB1-crystallin aggregation and degradation by separating two interacting loops in C-terminal domain

**DOI:** 10.1007/s13238-016-0284-3

**Published:** 2016-06-18

**Authors:** Liang-Bo Qi, Li-Dan Hu, Huihui Liu, Hai-Yun Li, Xiao-Yao Leng, Yong-Bin Yan

**Affiliations:** State Key Laboratory of Membrane Biology, School of Life Sciences, Tsinghua University, Beijing, 100084 China

**Keywords:** β/γ-crystallin, cataract-causing mutation, hydrophobic core, protein aggregation, protein folding

## Abstract

β/γ-Crystallins are predominant structural proteins in the cytoplasm of lens fiber cells and share a similar fold composing of four Greek-key motifs divided into two domains. Numerous cataract-causing mutations have been identified in various β/γ-crystallins, but the mechanisms underlying cataract caused by most mutations remains uncharacterized. The S228P mutation in βB1-crystallin has been linked to autosomal dominant congenital nuclear cataract. Here we found that the S228P mutant was prone to aggregate and degrade in both of the human and *E*. *coli* cells. The intracellular S228P aggregates could be redissolved by lanosterol. The S228P mutation modified the refolding pathway of βB1-crystallin by affecting the formation of the dimeric intermediate but not the monomeric intermediate. Compared with native βB1-crystallin, the refolded S228P protein had less packed structures, unquenched Trp fluorophores and increased hydrophobic exposure. The refolded S228P protein was prone to aggregate at the physiological temperature and decreased the protective effect of βB1-crystallin on βA3-crystallin. Molecular dynamic simulation studies indicated that the mutation decreased the subunit binding energy and modified the distribution of surface electrostatic potentials. More importantly, the mutation separated two interacting loops in the C-terminal domain, which shielded the hydrophobic core from solvent in native βB1-crystallin. These two interacting loops are highly conserved in both of the N- and C-terminal domains of all β/γ-crystallins. We propose that these two interacting loops play an important role in the folding and structural stability of β/γ-crystallin domains by protecting the hydrophobic core from solvent access.

**Electronic supplementary material:**

The online version of this article (doi:10.1007/s13238-016-0284-3) contains supplementary material, which is available to authorized users.

## Introduction

The vertebrate lens is a delicate machine with the ability to transmit and focus visible lights on the retina. The optical functions of the lens are achieved by the three-dimensional packing of the highly differentiated lens fiber cells and the unique protein expression profile in lens cells (Benedek, 1971; Bloemendal et al., 2004). In mature lens fiber cells, all organelles have been degraded via an apoptic-like pathway to avoid light scattering (Bassnett, 2009). The lack of protein turnover machineries implies that the lens proteins are required to maintain their solubility and stability for several decades (Bloemendal et al., 2004). Once the lens proteins precipitate into large aggregates, the transmission of visible lights will be scattered, which will lead to cataract (Benedek, 1997). Cataract, a typical protein aggregation disease, is one of the leading causes of human blindness worldwide. Similar to the other protein aggregation diseases, there are no clinical drugs available for cataract prevention and treatment. Currently surgical treatment is the only available method to cure both congenital and aged cataracts. Very recently, the findings of crystallin aggregation reversed by lanosterol (Zhao et al., 2015) and regeneration of human lens using endogenous stem cells of infants (Lin et al., 2016) provide new insights in the developing of novel strategies for cataract prevention and treatment.

The common feature of congenital and aged cataracts is the abnormal aggregation of various crystallins (Benedek, 1997; Moreau and King, 2012), which are the predominant soluble fractions in mature lens fiber cells (Bloemendal et al., 2004). According to the difference in their molecular sizes, structures and functions, crystallins can be classified into two superfamilies: α- and β/γ-crystallins (Bloemendal et al., 2004). α-Crystallins are small heat shock proteins and can function as molecular chaperones to inhibit the misfolding and aggregation of β/γ-crystallins (Horwitz, 1992). α-Crystallins can also protect lens cells against apoptosis (Morozov and Wawrousek, 2005). β/γ-Crystallins are the major structural proteins in the cytoplasm of lens fiber cells. There are seven members in human β-crystallins (βB1, βB2, βB3, βA1, βA2, βA3 and βA4) and five members in human γ-crystallins (γA, γB, γC, γD and γS). These β- and γ-crystallins share a conserved fold in their tertiary structures, but differ in their oligomeric sizes (Bloemendal et al., 2004). β-Crystallins are homomers or heteromers with molecular sizes ranging from 2 mer to 8 mer, while γ-crystallins are monomers (Bloemendal et al., 2004). Compared with γ-crystallins, β-crystallins generally possess longer N- and C-terminal extensions (Berbers et al., 1983; David et al., 1996), which are believed to be important to regulate the oligomerization and folding of β-crystallins (David et al., 1996; Leng et al., 2014; Dolinska et al., 2009; Sergeev et al., 1998; Kroone et al., 1994). Crystallins have been shown to not only function as structural proteins but also involve in lens development (Andley, 2007). Numerous mutations in crystallins have been identified to cause autosomal dominant congenital cataracts (ADCCs) and the list of mutations is increasingly risen every year (Shiels and Hejtmancik, 2015).

Each polypeptide of β/γ-crystallins is composed of four Greek-key motifs divided into two domains, the N- and C-terminal domain (NTD and CTD, respectively) (Bax et al., 1990). The hydrophobic core of each domain is formed by two Greek-key motifs and therefore the integrity of these Greek-key motifs is important to the structure and stability of β/γ-crystallins. Mutations of key residues involved in Greek-key motif formation in γD have been associated with more severe cataracts than those occurred at the loops, termini or surfaces of β/γ-crystallins (Vendra et al., 2013). Frameshift or nonsense mutations in β/γ-crystallins usually result in the loss of protein segments and thereby block the hydrophobic core formation. Besides acted as Greek-key motif breakers, mutations in β-crystallins may lead to cataract via complicated mechanisms such as changes in oligomerization and protein-protein interactions (Zhang et al., 2014; Xi et al., 2014a, b; Xu et al., 2010; Wang et al., 2013; Xu et al., 2012; Liu and Liang, 2005; Serebryany and King, 2014). Among the cataract-causing mutations in β-crystallins, there is a number of missense mutations occurred at the last β-strand of Greek-key motif 4. The last β-strand at the C-terminus of β-crystallins not only participates in the formation of the hydrophobic core, but also contributes to subunit interactions. Thus mutations occurred at the last β-strand may lead to cataract via quite different mechanisms. For example, V187E in βB2 is a Greek-key motif breaker, V187M in βB2 affects the hydrophobic core, while R188H in βB2 and R233H in βB1 affect the dynamic oligomeric equilibrium (Zhang et al., 2014; Xi et al., 2014a). At present it is difficult to predict how ADCC is caused by mutations in β-crystallins and experimental investigations are still necessary to elucidate the deleterious effects of these mutations.

 In this research, we studied the possible mechanism of ADCC caused by the S228P mutation in βB1. In 2007, S228P in βB1 was identified as a causive mutation of ADCC in a four-generation large Chinese family with nuclear cataract phenotype (Wang et al., 2007). S228 is fully conserved among various human β/γ-crystallins, but can be substituted by Ala in βB1 from the other vertebrate species (Fig. S1A). Structurally, S228 is the first residue of the last β-strand in Greek-key motif 4 and does not participate into the formation of monomer-monomer or dimer-dimer interfaces (Fig. S1B) (Montfort et al., 2003). Ser is a polar residue and thereby S228 is not a key residue of the hydrophobic core. To elucidate how βB1 structure and stability are affected by the S228P mutation, we compared the behaviors of the wild type (WT) βB1 and the S228P mutant at the protein and cellular levels. Our results showed that S228 stabilized two interacting loops to shield the CTD hydrophobic core from solvent access. The S228P mutation separated the two interacting loops from each other and allowed water molecules to penetrate into the hydrophobic core. More importantly, the positions and interaction of these two loops are highly conserved in various β/γ-crystallins, suggesting that the shielding effect of these two loops is important to the folding and stability of β/γ-crystallin domains.

## Results

### The S228P mutation promotes βB1 aggregation and degradation in both human and *E. coli* cells

**Figure 1:**
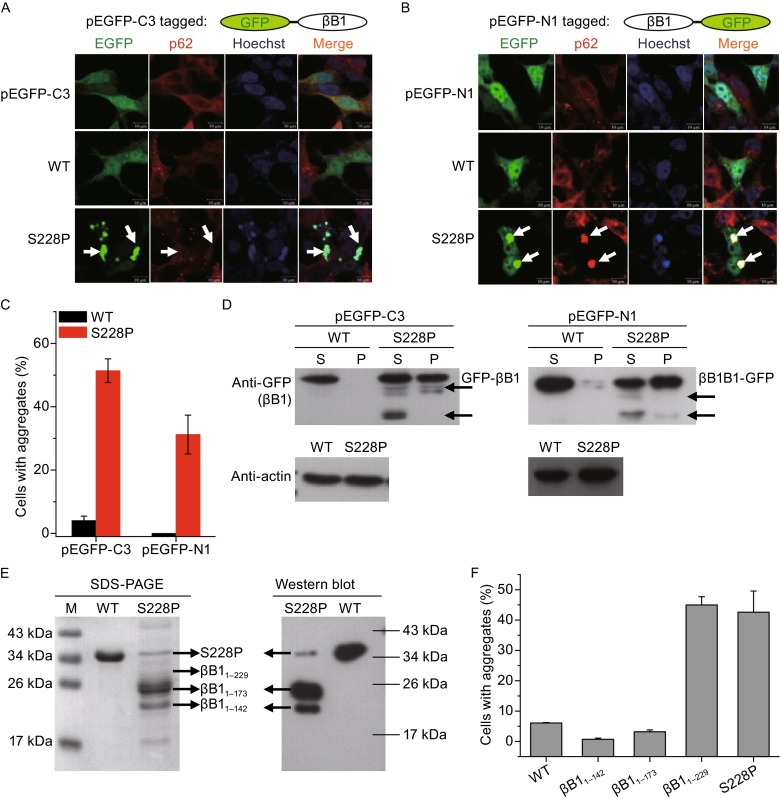
**The S228P mutation promoted βB1 aggregation and degradation in human HEK 293T cells and** ***E. coli*** **BL21 cells**. (A) Representative confocal images of HEK 293T cells with exogenously expressed WT βB1 and S228P fused by EGFP at the N-terminus. The empty vector of pEGFP-C3 was also transfected and used as the control. The βB1 proteins were visualized by the tagged GFP (green). The aggresomes were recognized by the marker protein p62 (red). The nucleus was stained by Hoechst 33342 (blue). The white arrows indicate the typical aggregates formed by GFP-S228P, which did not colocalize with p62. (B) Representative confocal images of HEK 293T cells expressing WT βB1 and S228P fused by EGFP at the C-terminus. The white arrows indicate the typical p62-positive aggregates formed by S228P-GFP. (C) Quantitative analysis of protein aggregation in HEK 293T cells. The percentages were obtained by calculating the ratios of cells with aggregates in ten random viewing field and the presented data were from three independent experiments. (D) Western blot analysis of the exogenously expressed WT βB1 and S228P in the HEK 293T cells. Actin was used as the loading control. The black arrows indicate the appearance of protein degradation. (E) SDS-PAGE and Western blot analysis of the WT βB1 and S228P overexpressed in *E. coli* BL21 cells. The proteins were purified by Ni-column and Superdex column. The degraded segments were identified by mass spectrometry. The Western blot analysis was performed using antibody against His-tag. (F) The proteolytic fragment βB1_1–229_ was prone to form aggregates in HEK 293T cells, while βB1_1–142_ and βB1_1–173_ were not. Representative confocal images are shown in Fig. S6

Previously we have shown that the phenotype of various mutated crystallins is independent on cell lines but dependent on the intrinsic property of the mutated proteins (Zhao et al., 2015). The WT βB1 and S228P mutant were exogenously expressed in human cell line HEK 293T and *E. coli* BL21 to evaluate whether the studies at the cellular and protein levels could mimic the ADCC phenotype (Fig. 1). For studies in the HEK 293T cells, GFP was fused to either the N- or C-terminus of the target protein (GFP-βB1 and βB1-GFP, respectively) to exclude misleading results caused by the introduction of the GFP tag. The WT βB1 showed the same disperse distribution pattern as the GFP controls, which was distributed in both the cytosol and nucleus of the HEK 293T cells (Fig. 1A and 1B). The position of the GFP tag does not affect the cellular distribution of βB1. The S228P mutant was prone to form intracellular aggregates for both types of fused proteins, while cells expressing GFP-S228P had a larger portion of cells containing aggregates than cells expressing S228P-GFP (Fig. 1C). The difference in percentages of cells with aggregates was not caused by the expression levels of the two types of fused proteins (Fig. S2). It is worth noting that GFP-WT also had a larger percentage of cells with aggregates than WT-GFP. A possible explanation is that the N-terminal extension has been shown to assist the folding of βB1 (Leng et al., 2014) and thereby the N-terminal tagged GFP might interfere with the function of the N-terminal extension. The major difference between the two types of fused proteins was their colocalization with p62, an aggresome marker. The aggregates formed by S228P-GFP were p62-positive, while those by GFP-S228P were p62-negative. Most crystallin mutants formed p62-positive aggresomes in human cells (Zhao et al., 2015; Xi et al., 2015). We further confirmed that GFP-S228P formed p62-negative aggregates by fusing S228P by a Flag tag at the N-terminus (Fig. S3). The absolute number of percentage of cells with Flag-S228P aggregates was lower than that of GFP-S228P, which might be caused the by lower expression level of Flag-S228P in the cells or inaccessibility of the Flag tag buried in the aggregates. Nonetheless, the results showed that the formation of p62-negative aggregates was independent on the type of tag fused at the N-terminus of S228P. Previously, the p62-positive crystallin aggregates have been shown to induce cell death. Here we found that the p62-negative S228P aggregates could also be toxic to the cells and induce cell apoptosis and necrosis (Fig. S3).

The exogenously expressed proteins were analyzed by Western blot using antibody against GFP (Fig. 1D). Consistent with the results from confocal microscope observations, Western blot analysis indicated that the WT proteins were predominantly in the supernatant fraction of cell lysates, while the mutated proteins were in both of the supernatant and precipitation fractions. There were S228P proteolytic segments that could be pulled down by the GFP antibody, while no proteolysis was observed for the WT proteins. GFP-S228P was more susceptible to degradation than S228P-GFP, which might be caused by the protective effect of the tagged GFP at the C-terminus. The proteolysis-prone GFP-S228P had higher propensity to form intracellular aggregates (Fig. 1C), suggesting that proteolysis might increase the aggregatory propensity of the protein.

The N-terminal His-tagged WT βB1 could be successfully obtained from the supernatant fractions of *E. coli* cells cultivated at 37°C (Fig. S4A). However, most of the S228P mutated proteins existed in the inclusion bodies when using the same induction and cultivating conditions as those for the expression of WT protein. When the *E. coli* cells were cultivated at 12°C, a small fraction of recombinant S228P proteins was in the soluble fractions and could be purified by Ni-column (Fig. S4B). However, little S228P proteins with the predicted molecular size could be obtained after purification by gel filtration chromatography at 4°C even though there were no halts during purification (Figs. 1E and S4B). Western blot analysis using antibody against His indicated that the purified proteins were His-tagged recombinant proteins (Fig. 1E). Mass spectrometry analysis of the major bands separated by SDS-PAGE indicated that the purified soluble proteins were N-terminal proteolytic segments of βB1, while the proteins in the inclusion bodies were dominated by intact S228P. These observations indicated that the behaviors of the S228P mutant in the *E. coli* cells were similar to those in HEK 293T cells, implying that the high susceptibility to aggregation and degradation was an intrinsic property of the S228P mutated βB1. To study whether proteolysis affect S228P aggregation, three N-terminal fragments, βB1_1–142_, βB1_1–173_ and βB1_1–229_ were constructed and overexpressed in the *E. coli* cells. Part of the recombinant βB1_1–142_ and βB1_1–173_ proteins existed in the supernatant fractions, while most of βB1_1–229_ proteins were in the inclusion bodies (Fig. S5). In the HEK 293T cells, most cells expressing βB1_1–142_ and βB1_1–173_ did not contain protein aggregates, while about 45% cells expressing βB1_1–229_ with a GFP tag at the N-terminus contained p62-negative aggregates (Figs. 1F and S6). This suggested that βB1_1–229_ was the main aggregation-prone proteolytic fragment.

**Figure 2:**
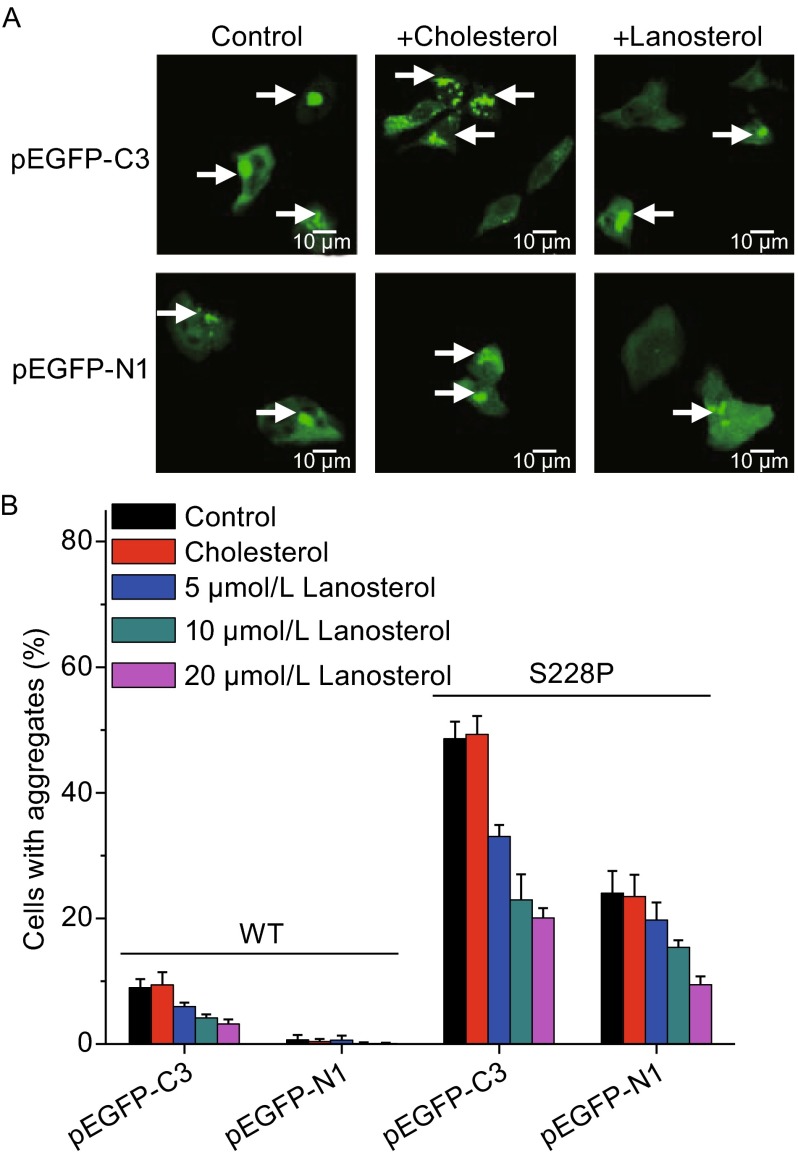
**S228P aggregates could be redissolved by lanosterol but not cholesterol in a concentration-dependent manner**. (A) Representative confocal images of cells expressing S228P treated with, 20 µmol/L cholesterol or lanosterol. For clarity, the staining of the nucleus was not shown. (B) Quantitative analysis of the percentage of cells with protein aggregates. The cells were transfected by plasmids containing the WT and mutated βB1 genes and cultivated for 24 h to allow the formation of crystallin aggregates. Then the cells were treated by 1% DMSO, cholesterol in 1% DMSO or lanosterol 1% DMSO

Recently, it has been identified that lanosterol, a sterol intermediate in the cholesterol synthetic pathway, can reverse crystallin aggregation (Zhao et al., 2015). The HEK 293T cells containing exogenously expressed WT and mutated βB1 were treated by various concentrations of lanosterol or cholesterol. The results herein confirmed the previous finding that lanosterol but not cholesterol could decrease the percentage of cells with crystallin aggregates in a concentration-dependent manner (Fig. 2). Lanosterol was effective to redissolve preformed aggregates for both types of fused proteins, implying that the action of lanosterol was independent on the nature of crystallin aggregates, p62-positive or p62-negative.

### The S228P mutation modifies βB1 refolding pathway

**Figure 3:**
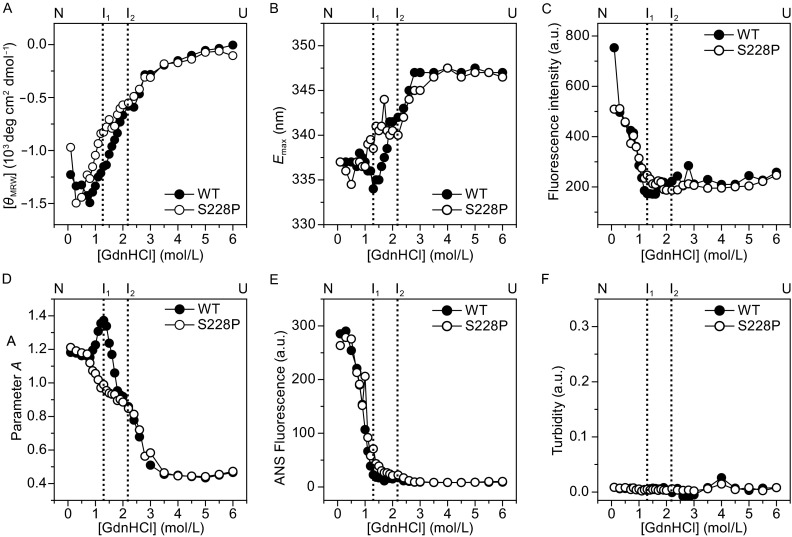
**The S228P mutation modified βB1 refolding pathway**. (A) Refolding monitored by ellipticity at 222 nm. (B) Refolding monitored by *E*_max_ of Trp fluorescence. (C) Refolding monitored by Trp fluorescence intensity. (D) Refolding monitored by Parameter *A* of Trp fluorescence spectra. (E) Refolding monitored by ANS fluorescence intensity at 470 nm. (F) Changes in turbidity during equilibrium refolding. The dotted lines indicate the positions where the refolding intermediates were populated

The results in Fig. 1 suggested that the S228P mutation might affect the folding of βB1 in human and *E. coli* cells. Equilibrium refolding of the GdnHCl-denatured proteins was performed to verify this proposal (Fig. 3). Similar to previous observations (Wang et al., 2011a), the refolding of βB1 was a multi-state process with the appearance of at least two equilibrium refolding intermediates (2U → 2I_2_ → I_1_ → N_2_). As proposed previously, I_2_ at 2.2 mol/L GdnHCl is a partially folded monomeric intermediate, while I_1_ at ~1.3 mol/L is a dimeric intermediate. I_1_ may be a state with extra shielding of the Trp fluorescence as evidenced by the blue-shift of the maximum emission wavelength (*E*_max_) or larger Parameter *A* value when compared to the native state (Wang et al., 2011a). Thus the transition from I_1_ to N involves structural arrangements around the Trp fluorophores. The transition curves from Trp fluorescence intensity, ANS fluorescence and turbidity were almost superimposed for the WT and S228P proteins. As for the transition curves from CD, *E*_max_ and Parameter *A*, no significant difference was observed for the transition from U to I_2_, while large deviations between the two proteins were found for the I_2_ → I_1_ transition. I_1_ of S228P had different structural features from those of WT βB1. Compared with I_1_ of WT βB1, I_1_ of S228P had less regular secondary structures, a red-shift of *E*_max_ and a smaller Parameter *A* value, suggesting that the S228P mutation retarded the formation of a well-packed dimeric intermediate.

### The refolded S228P mutant is unstable and prone to aggregate

**Figure 4:**
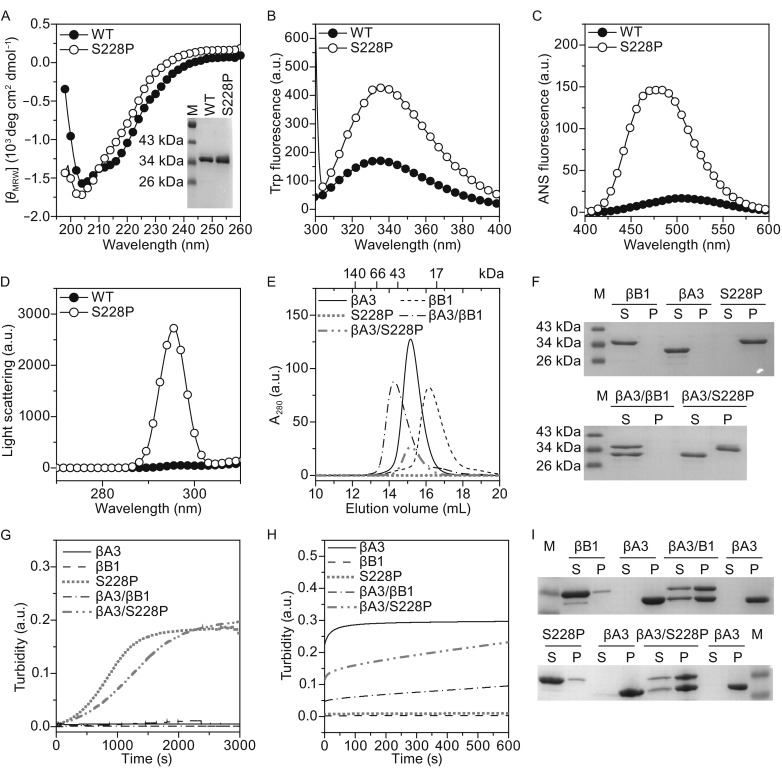
**Biophysical characterization of the refolded S228P proteins**. (A) Far-UV CD spectra. The CD signal is expressed in mean residue molar ellipticity ([*θ*_MRW_]). The inset shows SDS-PAGE analysis of the purified βB1 and the refolded S228P proteins. (B) Intrinsic Trp fluorescence spectra excited by 295 nm light. (C) Extrinsic ANS fluorescence spectra excited by 380 nm light. (D) Resonance Raleigh light scattering excited by 295 nm light. (E) SEC analysis of heteromer formed at 37°C. No UV absorption signals could be detected for the injected solutions of refolded S228P after centrifugation to remove protein aggregates. The UV absorption of βA3/S228P is lower than that of βA3/βB1. These unusual behaviors were caused by the precipitation of the refolded S228P. (F) SDS-PAGE analysis of the soluble and insoluble fractions of protein solutions incubated at 37°C for 1 h. (G) Aggregation kinetics monitored by changes in turbidity for protein solutions incubated at 37°C. The kinetic parameters were obtained by fitting the raw data using a first-order aggregation kinetics and are shown in Fig. S5. (H) Aggregation kinetics during refolding of the 6 mol/L GdnHCl-denatured proteins. The refolding is initiated by fast manual mixing of denatured proteins in buffer A. (I) SDS-PAGE analysis of the refolded proteins. The protein samples were prepared by fast dilution of the denatured proteins in buffer A for 1 h

The S228P mutation probably affected the final step of βB1 dimer assembly and structural rearrangements (Fig. 3). Since the native state of S228P could not be obtained by purification of recombinant proteins, we purified the inclusion bodies from the *E. coli* cells and the proteins in the water-insoluble fraction were dissolved by buffer A containing 6 mol/L GdnHCl. The refolded S228P was successfully obtained by one-step dilution of the 6 mol/L GdnHCl denatured proteins in the refolding buffer (buffer A). The refolded S228P was prone to degrade and aggregate and thus fresh refolded samples were used for the biophysical measurements (Fig. 4). Compared with the WT protein, S228P possessed more disordered structures as revealed by the shape of far-UV CD spectra (Fig. 4A). The *E*_max_ of S228P was similar to that of the WT protein (Fig. 4B). However, S228P had a much larger Trp fluorescence intensity, implying that the mutation impaired the correct quenching of Trp fluorophores in native β/γ-crystallins (Chen et al., 2009; Chen et al., 2006; Kosinski-Collins et al., 2004). The refolded S228P had much larger ANS fluorescence than the WT βB1 (Fig. 4C), suggesting that the refolded S228P failed to bury the hydrophobic side chains in the interior of the molecule. The oligomeric status of refolded S228P could not obtained by static light scattering due to fast degradation and aggregation of the proteins. Thus the molecular size of refolded S228P was qualitatively evaluated by resonance Raleigh light scattering, which is a sensitive monitor of change in molecular size of soluble oligomers (He et al., 2009). The refolded S228P protein had a much higher light scattering intensity than the WT protein (Fig. 4D), implying that the refolded S228P either had more disordered regions or had a larger oligomeric size.

In the lens, β-crystallins are proposed to form homomers or heteromers (Slingsby and Bateman, 1990). In the βA/βB heteromers, βB-crystallins are thought to protect the relative instable βA-crystallins against aggregation (Leng et al., 2014; Wang et al., 2011a, b; Bateman et al., 2001; Bateman et al., 2003). The heteromers were formed by mixing equal molar of homomers and incubated at 37°C as described previously (Wang et al., 2011a). SEC (Fig. 4E) and SDS-PAGE analysis (Fig. 4F) were used to characterize heteromer formation. As the control, the WT βB1 and βA3 prefer to exist as heterodimer after incubation. However, almost all molecules of refolded S228P precipitated and little βA3/S228P heteromers could be obtained. To elucidate whether refolded S228P lost its ability to associate with βA3 or aggregated before association, we monitored the time-course change in turbidity after mixing βA3 and S228P at 37°C. As the controls, βA3, βB1 and βA3/βB1 were stable at the physiological temperature. However, refolded S228P aggregated rapidly at 37°C (Fig. 4G). The addition of βA3 could elongate the lag time and slow down the rate of S228P aggregation, but did not decrease the extent of aggregation (Fig. S7). Thus it seems that the association between βA3 and S228P could occur but was greatly weakened by the mutation. To confirm this proposal, the 6 mol/L GdnHCl-denatured S228P were refolded in the presence or absence of βA3 (Fig. 4H and 4I). Similar to previous results (Leng et al., 2014; Wang et al., 2011a), the WT βB1 could associate with βA3 during co-refolding and protect βA3 against misfolding and aggregation. The S228P mutant did not aggregate during refolding from the GdnHCl-denatured state, but partially lost the protective effect on βA3. Thus the S228P mutation weakened the ability of βB1 to form heteromers with βA3, probably by perturbation of the correct refolding of βB1.

### The S228P mutation separates two interacting loops that shield the hydrophobic core of the C-terminal domain from solvent

**Figure 5:**
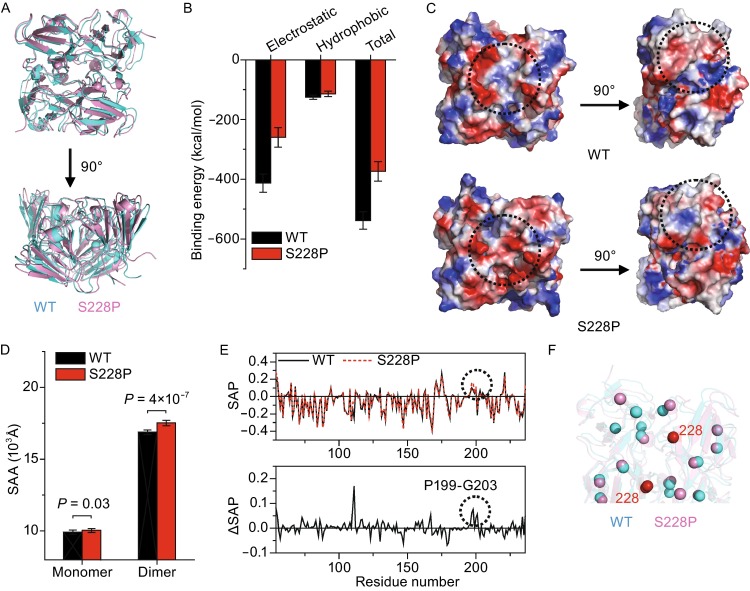
**The S228P mutation weakened subunit binding interface and modified surface charge distributions**. (A) A comparison of the simulated dimeric structures of the WT βB1 and S228P. (B) Average subunit binding energies calculated from the simulated structures. The time-course changes in subunit binding energies are shown in Fig. S7. (C) Surface electrostatic potentials of the dimeric βB1 and S228P. The S228P mutation altered the distribution of charged residues and the representative areas are indicated by dotted circles. (D) Solvent accessible areas (SAA) of the simulated monomeric and dimeric structures. (E) Spatial aggregatory potential (SAP) of the WT and mutated proteins. The extra exposed residues in the mutant were selected by an increase of SAP for residues with positive SAP values. The dotted circle indicates the region with the largest change in SAP. (F) Positions of Trp fluorophores in the simulated dimeric structures of βB1 and S228P. The Cα atoms of Trp residues are shown in spheres. The positions of residue 228 are also shown in red

S228 is the first residue forming the last β-strand of Greek-key motif 4 in βB1 (Fig. S1B). Ser has a polar side chain and consequently S228P is not a key residue in the formation of the hydrophobic core. MD simulations were performed for both the dimeric and monomeric (subunit A of the dimer) structures to explore the structural basis for the deleterious effects of the S228P mutation on βB1 structure, folding, assembly and stability. The structural variations for all simulated proteins were within 3 Å and the simulations could reach equilibrium after ~7.5 ns (Fig. S8A). Under our simulating conditions, no significant difference was observed between the simulated dimeric and monomeric structures (Fig. S8B). Alignment of the dimeric structures of βB1 and S228P indicated that the mutation did not alter the overall fold of βB1 (Fig. 5A). However, the S228P mutation greatly reduced the subunit binding energy arisen from electrostatic interactions (Figs. 5B and S9A). It has been proposed that the subunit interface is mainly stabilized by the H-bonding network among two acidic residues (D169 and D170) and two basic residues (R231 and R233) in each subunit (Montfort et al., 2003). The H-bonding network was maintained in the subunit interface of S228P, but the positions of these charged residues were slightly altered in the simulated S228P dimeric structure (Fig. S9B). A close inspection of the surface electrostatic potentials indicated that the S228P mutation altered the distribution of charged/polar residues around the subunit interface (Fig. 5C). The mutation did not significantly alter the solvent accessible surface area (SAA) of the subunit, but increased the SAA of the dimer (Fig. 5D), which further suggested that decrease in subunit binding energy might result in the exposure of more buried residues in the interface to solvent.

Although a significant increase in ANS fluorescence was observed for the refolded S228P protein (Fig. 4C), it seems that the overall hydrophobic exposure was not significantly affected by the S228P mutation when analyzing the surface electrostatic potentials of the simulated dimeric and monomeric structures (Figs. 5C and S10). The main difference between the WT and mutated proteins was the distribution of surface charged/polar regions and the hydrophobic regions. Thus a possible explanation for the increased ANS fluorescence shown in Fig. 4C is that the mutated protein might contain more hydrophobic sites accessible by the ANS probes. A comparison of the spatial aggregation propensity (SAP) values (Chennamsetty et al., 2009) indicated that the major difference between the two proteins was at the region, ^199^PGYRG^203^ (Fig. 5E). The positions of most Trp residues except for W237 at the C-terminal extension were not significantly affected by the mutation (Fig. 5F), which is consistent with the unchanged *E*_max_ of Trp fluorescence (Fig. 4B). The mutation-induced increase of Trp fluorescence was more likely to be caused by the change in the rigidness of the hydrophobic core or quenching of Trp fluorescence by adjacent residues.

**Figure 6:**
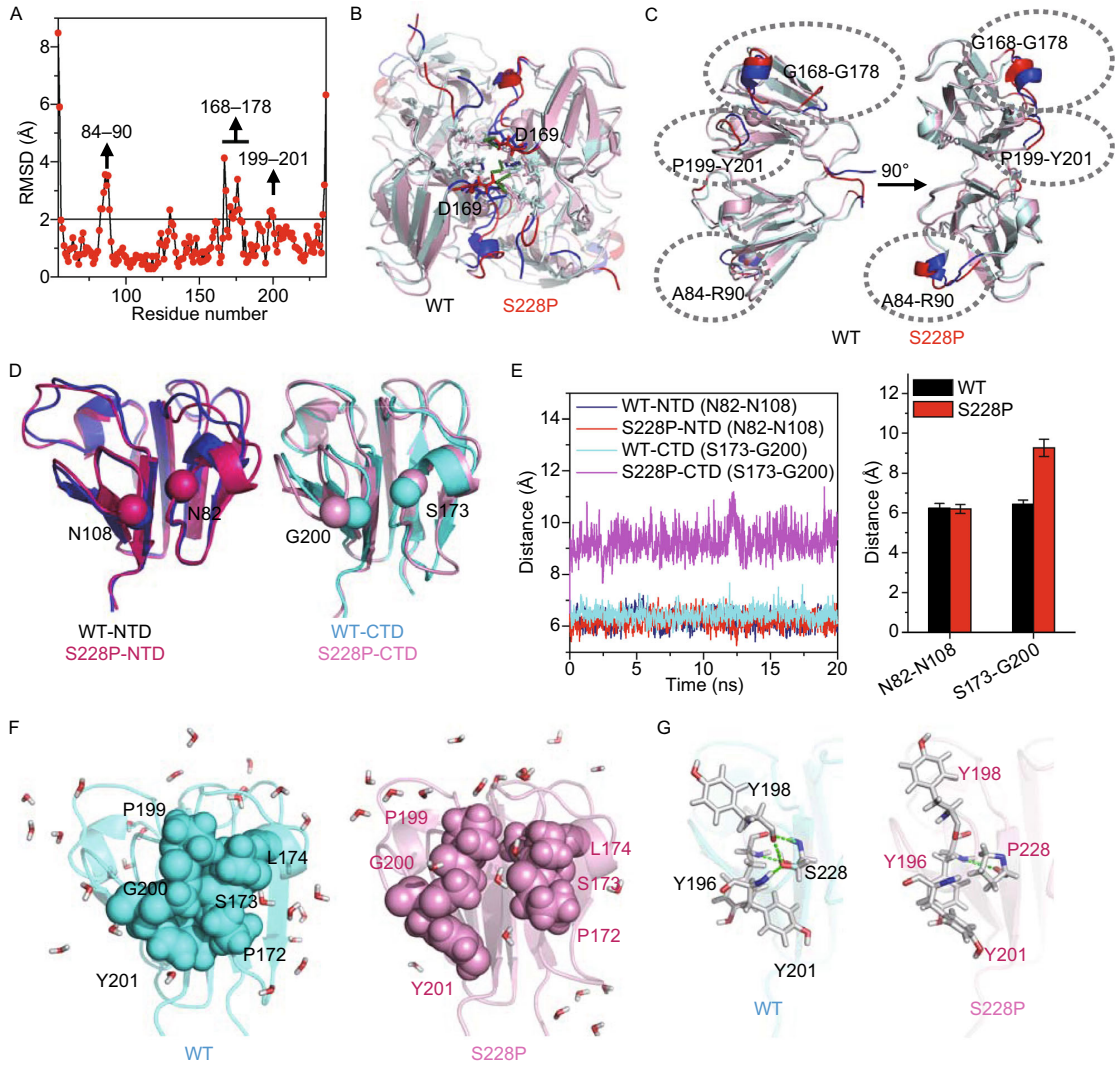
**The S228P mutation separated the two interacting loops in the C-terminal domain of βB1**. (A) Structural variations between the WT βB1 and S228P. RMSD values were calculated from the last frame of the simulated structures. The residues with significant difference were identified to have a RMSD value larger than 2 Å. Time-course changes in structural variations during simulation are presented in Fig. S9A. (B) Positions of residues with RMSD value larger than 2 Å in the dimeric structures. (C) Positions of residues with RMSD value larger than 2 Å in the subunit structures. The three regions with highest variations are indicated by dotted circles. (D) The hydrophobic core of the structurally conserve NTD and CTD is covered by two interacting loops. The S228P mutation separated the two loops in CTD but not those in NTD. The Cα atoms of residues used for distance determination are highlighted by spheres. (E) Distance between the two loops determined from the Cα atoms between N82 and N108 in NTD and their equivalent residues S173 and G200 in CTD. The left panel shows the time-course changes in the distances and the right panel shows the average value of the distances. (F) Open of the interacting loops in S228P allowed water molecules to penetrate in the hydrophobic core of CTD. The water molecules are shown in stick model. (G) S228 formed H-bonding network with three Try residues and stabilize the interacting loops, which is disrupted by the S228P mutation

The major structural changes in the subunit structure caused by the S228P mutation was obtained by tracing the structural fluctuations during the 20 ns simulations (Fig. S11A). Two adjacent interacting loops, P199-G203 and P172-V176, had large variations in S228P but not in WT βB1 (Fig. S11B). Similar results could also be obtained by comparing the RMSD values of the final frame of the simulated structures (Fig. 6A) except for the identification of an additional region ranging from A84 to R90 in NTD, which is equivalent to the G168 to G178 region in CTD (Fig. 6B and 6C). It is worth noting that D169 is a key residue forming subunit interface (Figs. 5B and S9B) (Montfort et al., 2003). Structurally, most parts of these three regions with the largest structural changes formed loops and surface helices to cover the hydrophobic core of NTD and CTD. The movement of the A84 to R90 region did not affect the positions of the two interacting loops in NTD, whereas the closely packed loops in CTD were separated from each other in the simulated structures of S228P (Fig. 6D). The distances between the two interacting loops provided a quantitative evaluation of separation (Fig. 6E). The distance between the two loops was about 6.2 Å in both NTD and CTD of the WT βB1 as well as NTD of S228P, but separated by 9.3 Å in CTD of S228P. The separation of the two interacting loops by the S228P mutation allowed the solvent molecules to penetrate in the hydrophobic core of CTD (Fig. 6F) and thereby might retard CTD dehydration during refolding. In WT βB1, S228 helps to tether the P199-Y201 loop by forming H-bonds with Y196, Y198 and Y201 via its side-chain OH and backbone NH, which were disrupted by the S228P mutation (Fig. 6G). The above structural analysis suggested that the S228P mutation did not perturb the hydrophobic core directly, but influenced the protection of the hydrophobic core from solvent by the two interacting loops.

## Discussion

Cataract is a prevalent disease that is directly caused by the appearance of light-scattering crystallin aggregates (Benedek, 1997; Moreau and King, 2012). Although crystallin aggregation is a common feature of various cataracts, the ways of highly soluble crystallins to aggregation may be very complicated (Vendra et al., 2013; Zhang et al., 2014; Xi et al., 2014a, b; Xu et al., 2010; Wang et al., 2013; Xu et al., 2012; Liu and Liang, 2005; Serebryany and King, 2014). Dissection of the mechanisms underlying various cataracts will facilitate the development of potential strategies for cataract prevention and treatment. Meanwhile, crystallins have been taken as model proteins in the protein folding field for a long time. Numerous cataract-causing crystallin mutations also provide invaluable information to study the structural determinants of protein folding and aggregation. Previous studies have shown that the integrity of the Greek-key motifs is important to the understanding of β/γ-crystallin folding and misfolding (Vendra et al., 2013; Moreau and King, 2009; Flaugh et al., 2005). This proposal was also reinforced by our results. The mutation at S228, which is the first residue at the last β-strand of Greek-key motif 4, resulted in severe changes in βB1 folding and stability. Our results showed that the mutated protein could not gain the native structure of βB1. The misfolded S228P protein was prone to aggregate and degrade in both human and *E. coli* cells. Our results strongly suggested that S228 was an essential structural residue for βB1 folding.

**Figure 7:**
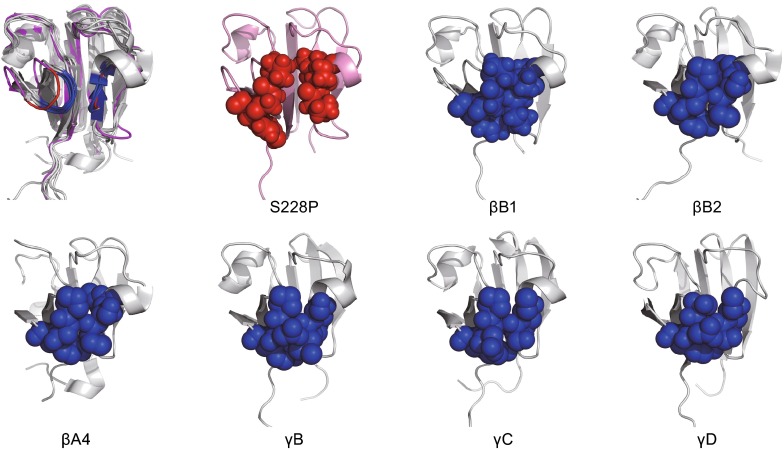
**The two interacting loops in the C-terminal domain of βB1 are conserved in various β/γ-crystallins**. The upper left panel shows superimposed structures of CTD from βB1 (PDB ID: 1OKI), βB2 (PDB ID: 1BLB), βA4 (PDB ID: 3LWK), γB (PDB ID: 2JDF), γC (PDB ID: 2V2U) and γD (PDB ID: 1HK0). The loops of the WT proteins are shown in blue and those of S228P are in red. The sphere models show that the interaction between the two loops is conserved across various β/γ-crystallins. The structurally equivalent loops could also be found in NTD of various β/γ-crystallins (Data not shown)

Unlike the other mutations occurred at residues in the conserved Greek-key motifs, the S228P mutation did not disrupt the hydrophobic core directly. According to the structural studies by MD simulations, we propose that the hydrophobic core of βB1 CTD is protected by surface loops and helices, particularly gated by two interacting loops formed by residues around S173 and G200. Alignment of NTD and CTD structures indicated that NTD also possessed such two gating loops. The distance between the backbones of these two loops was the same for NTD and CTD though the composing residues are not conserved in their primary sequences. The movements of these two loops in WT βB1 were highly restricted but had higher fluctuations in S228P. Structurally, these two loops acted as the “doors” to protect the hydrophobic core. The closure of the doors prevented solvent molecules to access the hydrophobic core and thereby stabilized the dehydrated core structure (Fig. 6F). S228 locked the doors by stabilizing the loop around G200 via forming H-bonding network with Y196, Y198 and Y201. Considering that this Ser can be substituted by Ala in some species (Fig. S1A), it seems that the polar side chain of Ser does not play a major role in S228 functions. The backbone of S228 forms two hydrogen bonds with Y196, which may be important to stabilize these two loops. Alignment of domain structures from various β/γ-crystallins indicated that the positions and interaction of these two loops are highly conserved in the β/γ-crystallin superfamily (Fig. 7). Therefore the shielding effect of these two interacting loops is probably a general structural factor governing the structural stability and folding of all β/γ-crystallin domains. Further research in the other β/γ-crystallins is needed to verity this proposal. It will be interesting to investigate whether the other globular domains also possess similar structures to shield the hydrophobic core from solvent access.

Globular proteins are characterized by the burial of apolar residues in the hydrophobic core and the solvation of surface polar residues (Baldwin, 2007; Prabhu and Sharp, 2005). Thus desolvation of residues forming the hydrophobic core is crucial to the folding of globular proteins. Based on our observations, we propose that the final step of domain folding in β/γ-crystallins is to close the doors formed by the two loops and thereby to accomplish the formation of a relatively dry core structure. Failure to close the doors, such as the disruption by the S228P mutation, will retard the completeness of domain folding. This proposal is supported by the significant deviation in the I_2_ to I_1_ transition of S228P from that of the WT βB1 (Fig. 3). Compared with I_2_ of βB1, I_2_ of S228P had an about 8 nm red-shift of the Trp fluorescence, indicating that the Trp fluorophores in I_2_ of S228P were more solvent accessible. Although the refolded S228P had a similar *E*_max_ value to WT βB1, the dramatically high Trp fluorescence intensity and ANS fluorescence intensity indicated that refolded S228P failed to bury all hydrophobic residues and the fluorescence quenching residues were wrongly positioned. Consequently, the refolded S228P was instable and prone to aggregate at the physiological temperature. It is worth noting that one of the proteolytic sites was around S173, which suggested that the gateless CTD in S228P was more prone to be attacked by water and trace amount of heavy metals or proteases. The susceptibility of S228P C-terminus to degradation also confirmed the proposal that the C-terminal domain was less folded in the S228P mutant.

In summary, we propose that the S228P mutation disrupted the interactions between the two gated doors of the hydrophobic core in CTD. Failure to shield the hydrophobic core from solvent led to a less packed C-terminal domain with extra hydrophobic exposure. The misfolded S228P mutant was prone to degrade and aggregate in the cells, which finally formed large light-scattering particles in the cataractous lens in patients. A possible method to prevent or cure the cataractous lens caused by the S228P mutation is to develop lanosterol-based drugs since lanosterol could redissolve intracellular S228P aggregates, no matter whether they were p62-positive or p62-negative.

## Materials and methods

### Materials

Ultra-pure guanidine hydrochloride (GdnHCl), sodium dodecyl sulfate (SDS), phenylmethylsulfonyl fluoride (PMSF), dithiothreitol (DTT), isopropyl-1-thio-β-D-galactopyranoside (IPTG) and 1-anilinonaphtalene-8-sulfonate (ANS) were purchased from Sigma. Dulbecco’s modified Eagle’s medium (DMEM), fetal bovine serum (FBS), lipofectamine, 2000, lipofectamine 3000 and Hoechst 33342 were purchased from Invitrogen. The antibody against p62 was obtained from Abcam, while the antibodies against green fluorescence protein (GFP) and β-actin were from Bioworld. Propidium iodide (PI) and annexin V were from BD Biosciences. The enzymes used for molecular cloning were from TaKaRa and the plasmid kits were from Tiangen. All other chemicals were local products of analytical grade.

### Protein sample preparation

Human *CRYBB1* and *CRYBA3* genes were cloned from the human lens cDNA library as described previously (Wang et al., 2011b). The S228P mutant of βB1 was obtained by site-directed mutagenesis using the following primers: For-βB1, 5′-CGGGATCCATGTCTCAGGCTGCAAAGGC-3′ and Rev-βB1, 5′-CCCAAGCTTTCACTTGGGGGGCTCTGTG-3′. The C-terminal truncated mutants of βB1 were constructed using the following primers: For-βB1_1–142_, 5′-ACATCATATGATGTCTCAGGCTGCAAAGGCCT-3′; Rev-βB1_1–142_, 5′-TGAAAGCTTTCAGATGGGCCGGAAGGACATGAGCCG-3′, For-βB1_1–173_, 5′-ACATCATATGATGTCTCAGGCTGCAAAGGCCT-3′; Rev-βB1_1–173_, 5′-TGAAAGCTTTCAACTGGGTGCGTCGTCCCCCTGGA-3′, For-βB1_1–229_, 5′-ACATCATATGATGTCTCAGGCTGCAAAGGCCT-3′; Rev-βB1_1–229_, 5′-TGAAAGCTTTCACAGGGGCTGCATCTGTGGCTGGA-3′. The cloned genes were inserted into the prokaryotic vector pET28a or the eukaryotic vectors pEGFP-C3, pEGFP-N1 and pcDNA-3.1 N-Flag for further exogenous expression in *E. coli* and human cells. Overexpression and purification of the His-tagged recombinant proteins in *E. coli* BL21 (DE3) were the same as the procedures described previously (Wang et al., 2011b). In brief, the overexpression of the recombinant proteins were induced by IPTG and the optimal IPTG concentration and cultivating temperature were screened from 0.05 mmol/L to 1 mmol/L for each protein. The IPTG concentrations and cultivating temperatures were 0.2 mmol/L and 37°C for βB1 and βA3, 0.1 mmol/L and 25°C for βB1_1–142_, βB1_1–173_ and βB1_1–229_, and 0.05 mmol/L and 12°C for S228P. The His-tagged proteins were extracted from the supernatant of the cell lysates with the addition of 1 mmol/L protease inhibitor PMSF and 1:1000 cock-tail proteases (Sigma), purified by Ni^2+^-affinity chromatography using a 1 mL Ni-NTA column. The contaminating proteins were washed using 40 mmol/L imidazole and the target proteins were obtained by 300 mmol/L imidazole. The final products were purified by gel filtration chromatography using a Superdex 200 preparation-grade column. The peaks in the elution profiles with purity above 95% were collected, concentrated by PEG20000 and stored at −80°C. The purity of the proteins was analyzed by 12.5% SDS-PAGE. The purified proteins were dissolved in buffer A (20 mmol/L sodium phosphate, 150 mmol/L NaCl, 1 mmol/L EDTA and 1 mmol/L DTT, pH 7.0). The protein concentration was determined using molar absorption coefficients of 1.819, 1.819, 1.164, 0.98 and 2.296 for βB1, S228P, βB1_1–142_, βB1_1–173_ and βA3, respectively. The βB1/βA3 heterodimer was prepared by incubating equal molar of βB1 and βA3 at 37°C for 3 h as described elsewhere (Wang et al., 2011a).

### Refolding of proteins from the inclusion bodies

Since the intact S228P proteins mainly existed in inclusion bodies when overexpressed in *E. coli* cells, the non-proteolytic S228P proteins were obtained by refolding from the inclusion bodies by dilution. The inclusion bodies were separated from the precipitation fractions of cell lysates in the presence of 1 mmol/L PMSF and washed by buffer B (20 mmol/L sodium phosphate, 500 mmol/L NaCl, 1 mol/L GdnHCl and 0.5% Triton-100, pH 7.0) for three times to remove the unexpected proteins and membranes. The purified inclusion bodies were dissolved in buffer C (20 mmol/L sodium phosphate, 500 mmol/L NaCl and 6 mol/L GdnHCl, pH 7.0). The His-tagged proteins in the inclusion bodies were purified by Ni^2+^-affinity chromatography and gel filtration chromatography using the same procedures as those for the purification of soluble proteins except that all buffers contained 6 mol/L GdnHCl. The purified 6 mol/L GdnHCl-denatured proteins were concentrated by PEG20000 to a final concentration of 20 mg/mL. Protein refolding was performed by diluting the 6 mol/L GdnHCl-denatured proteins in buffer A with a dilution ratio of 1:100. The refolding solutions were incubated at 4°C for 20 h and then the supernatant fractions were collected. The refolded proteins were further purified by gel filtration chromatography at 4°C and the peaks with >95% purity were collected, concentrated and stored at −80°C.

### Spectroscopic experiments

Far-UV circular dichroism (CD) was recorded on a Chirascan spectrophotometer using a 0.1 cm path-length cell. The samples for the far-UV CD experiments were in buffer D (2 mmol/L sodium phosphate, 15 mmol/L NaCl, 0.1 mmol/L EDTA and 0.1 mmol/L DTT, pH 7.0) to reduce the influence of high concentration of salts. The fluorescence spectra measured were on a Hitachi F-2500 spectrofluorimeter using a 1 cm path-length curvette. The intrinsic Trp fluorescence was excited by 295 nm light, while the extrinsic ANS fluorescence was excited by 380 nm light. The samples for ANS fluorescence measurements were prepared by adding 1:75 ANS probes to the protein solutions. Parameter *A*, which is a sensitive monitor of the changes in the shape and position of Trp fluorescence spectra (Turoverov et al., 1976), was calculated by dividing the fluorescence intensity at 320 nm by that at 365 nm (*I*_320_/*I*_365_). Resonance Raleigh light scattering was measured using an excitation wavelength of 295 nm as described previously (He et al., 2009). Turbidity was determined by the absorbance at 400 nm of the protein solutions on an Ultraspec 4300 pro UV/Visible spectrophotometer. Most spectroscopic experiments were performed using a protein concentration of 5.8 µmol/L (~0.2 mg/mL) unless otherwise indicated.

### SEC and SDS-PAGE analysis

The SEC analysis was performed using a Superdex 200 column equipped on an ÄKTA FPLC with an elution rate of 0.5 mL/min. About 100 µL protein solutions were injected in the column. The protein concentration for SEC analysis was 30 µmol/L (~1 mg/mL) and buffer A was used as the elution buffer. The SDS-PAGE analysis was performed using the 12.5% separating gel. About 40 µL protein solutions with a protein concentration of 0.2 mg/mL were used for the SDS-PAGE analysis.

### Protein refolding experiments

The fully denatured proteins were prepared by incubating the proteins in buffer A containing 6 mol/L GdnHCl at 25°C for 24 h. The equilibrium refolding of the proteins were performed by diluting the fully denatured proteins in buffers with various final concentrations of GdnHCl ranging from 0.1 mol/L to 6 mol/L at 4°C overnight. The transition curves of protein equilibrium refolding were obtained by monitoring the GdnHCl-dependence of changes in far-UV CD, intrinsic Trp fluorescence, extrinsic ANS fluorescence, light scattering and turbidity. The appearance of aggregates during kinetic refolding was measured by time-course change in turbidity after protein refolding initiated by 1:50 manual dilution of the 6 mol/L GdnHCl-denatured proteins into buffer A. The turbidity data were recorded every 2 s for 10 min. After 1 h refolding, the samples were centrifuged and analyzed by 12.5% SDS-PAGE. The final GdnHCl and protein concentration were 0.12 mol/L and 0.2 mg/mL, respectively.

### Protein aggregation kinetics at the physiological temperature

Aggregation kinetics at the physiological temperature was determined by incubating 0.2 mg/mL homomers or heteromers at 37°C for 50 min. The temperature was controlled by a water bath. The time-course change in turbidity was recorded on an Ultraspec 4300 pro UV/Visible spectrophotometer every 2 s during the incubation of protein solutions at 37°C. After 50 min incubation, the reaction was terminated on ice and was further analyzed by SEC and SDS-PAGE.

### Cell culture and immunofluorescence

The genes of the *CRYBB1* and *S228P* were constructed into the eukaryotic vector pEGFP-C3, pEGFP-N1 or pcDNA 3.1 N-Flag. The HeLa and HEK 293T cell lines were from ATCC. The recombinant plasmids were exogenously expressed in HEK 293T cells by transient transfection using lipofectamine 3000. The intracellular distribution of the exogenously expressed proteins was studied using the same procedure as those reported previously (Zhao et al., 2015; Xi et al., 2015). In brief, the 293T cells were cultured in the DMEM medium with the addition of 10% FBS at 37°C in 5% CO_2_ incubator. After transient transfection by lipofectamine 3000 for 6 h, the cells were transferred to fresh DMEM medium with the addition of 10% FBS and cultivated at 37°C for 24 h. Then the transfected cells were washed by PBS buffer for three times, fixed by 4% paraformaldehyde for 40 min, washed three times by PBS buffer, treated by 0.4% Trition X-100 for 10 min and blocked by 10% FBS for 40 min. The fixed cells were stained by dyes and antibodies, followed by washing three times using the PBS buffer. The nuclei were dyed with Hoechst 33342. The aggresomes were detected by the marker protein p62, which was recognized by Alexa 649-conjugated goat antibody against mouse p62. The exogenously expressed proteins were visualized by the tagged GFP. The distribution of the proteins in the cells was studied by a Carl Zeiss LSM 710 confocal microscope. The percentage of cells with aggregates was qualified by calculating the percentages from 200 positively transfected cells in ten randomly selected viewing fields. The mean fluorescence intensity of transfected cells was calculated by ImageJ (Collins, 2007).

### Cell apoptosis assay

The cell viability was determined by Cell Counting Kit-8 (CCK-8) using the same procedure as those reported previously (Xi et al., 2015). In brief, the HeLa cells were transiently transfected by the empty vector pcDNA 3.1 N-Flag and the recombinant plasmids containing the WT *CRYBB1* and *S228P* genes. The transfected cells were cultured at 37°C for 24 h and then were double stained by propidium iodide (PI) and annexin V. The percentages of apoptotic and necrotic cells were analyzed by bivariate flow cytometry on a FACSCalibur flow cytometer from BD Biosciences.

### Molecular dynamic simulations

Details of molecular dynamic (MD) simulation analysis were the same as those described previously (Wang et al., 2013; Chen et al., 2012). In brief, the starting structure of the mutant S228P was constructed by single site mutagenesis by PyMOL (The PyMOL Molecular Graphics System, Version 0.99rc6, Schrödinger, LLC, http://www.pymol.org/) using the crystal structure of truncated human βB1 (PDB ID: 1OKI) (Montfort et al., 2003) as the template. MD simulations were performed in a water box containing 150 mmol/L NaCl generated by Visual molecular dynamics (VMD) encoded in Nanosecond molecular dynamics (NAMD), version 2.9 (Phillips et al., 2005). After equilibration of the system for 5.8 ns, the simulations were run at 2 fs time-steps for 20 ns at 310 K and constant pressure (1 atm). The simulated structures were analyzed by VMD and PyMOL. Both dimeric structure and subunit structure (monomer) were applied to MD simulations to evaluate the effects of the S228P mutation on structures of the Greek-key motifs and dimer assembly.

## Electronic supplementary material

Below is the link to the electronic supplementary material.

Supplementary material 1 (PDF 1252 kb)
