# Supplementary material for: Cataract-causing mutation S228P promotes βB1-crystallin aggregation and degradation by separating two interacting loops in C-terminal domain
Source: Protein Cell. 2016 Jun 18;7(7):501–15. doi: 10.1007/s13238-016-0284-3 (PMC4930773; doi:10.1007/s13238-016-0284-3)
Supplement: Supplementary file 1 — Supplementary material 1 (PDF 1252 kb) [file 13238_2016_284_MOESM1_ESM.pdf]

Supplementary Materials

Cataract-causing mutation S228P promotes βB1-crystallin aggregation and degradation by separating two interacting loops in the C-terminal domain

Liang-Bo Qi[1], Li-Dan Hu[1], Huihui Liu, Hai-Yun Li, Xiao-Yao Leng, Yong-Bin Yan[2]

State Key Laboratory of Membrane Biology, School of Life Sciences, Tsinghua University, Beijing 100084, China

[1] These authors contributed equally to this work.

[2] To whom all correspondence should be addressed: Dr. Yong-Bin Yan, School of Life Sciences, Tsinghua University, Beijing 100084, China. Phone: +86-10-6278-3477; Fax: +86-10-6277-2245; E-mail: ybyan@tsinghua.edu.cn.

Short Title: S228P promotes βB1-crystallin aggregation and degradation

A

```
GA_HUMAN   166 SLRRVTDLY----------------
GB_HUMAN   167 SLRRVMDLY----------------
GC_HUMAN   166 SLRRVVDLY----------------
GD_HUMAN   166 SLRRVIDFS----------------
GS_HUMAN   172 SFRRIVS------------------
BA4_HUMAN  190 SIRRIQQ------------------
BA2_HUMAN  191 SIRRVQH------------------
BA1_HUMAN  209 SIRRIQQ------------------
BB3_HUMAN  193 SVRRIRDQKWHKRGRFPSS------
BB2_HUMAN  186 SVRRIRDMQWHQRGAFHPSN-----
BB1_HUMAN  228 SLRRLRDKQWHLEGSFPVLATEPPK
BB1_CHICK  215 SIRRIRDMQWDQKGTF-VTPEAPS
BB1_PIG    225 AVRRLRDRQWHREGCFPVLAAEPPK
BB1_BOVIN  229 AVRRLRDRQWHREGCFPVLAAEPPK
BB1_MOUSE  226 AVRRLRDRQWHQEGCFPVLTAEPPK
```

B

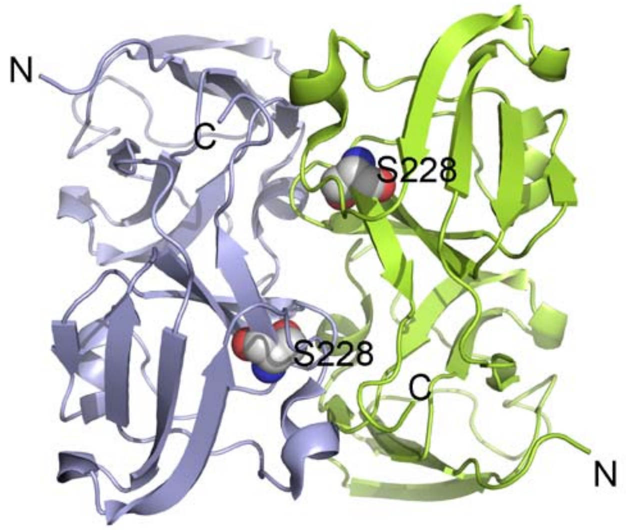

Supplementary Figure S1. S228 is the first residue of the last β-strand at the C-terminus. (A) Sequence alignment of the C-terminus of various human β/γ-crystallins and βB1 from various species. (B) Crystal structure of the truncated human βB1 (PDB ID: 1OKI). S228 is highlighted by the sphere model. N and C represent the N-terminus and C-terminus, respectively.

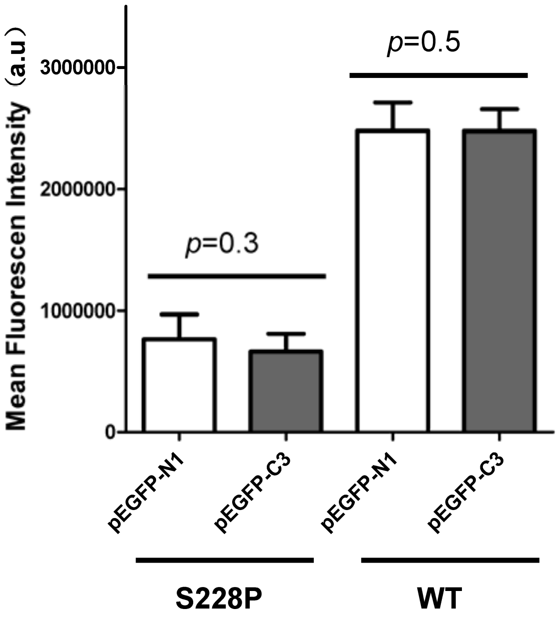

Supplementary Figure S2. A comparison of the expression level of proteins with GFP tagged at the N- or C-terminus. The mean fluorescence intensity was obtained by measuring the average fluorescence intensity of at least 10 transfected cells. The fluorescence intensity was determined by ImageJ. No significant difference was observed between the two types of fused proteins. The difference between the WT and mutated proteins was proximately caused by the degradation-prone property of the mutant.

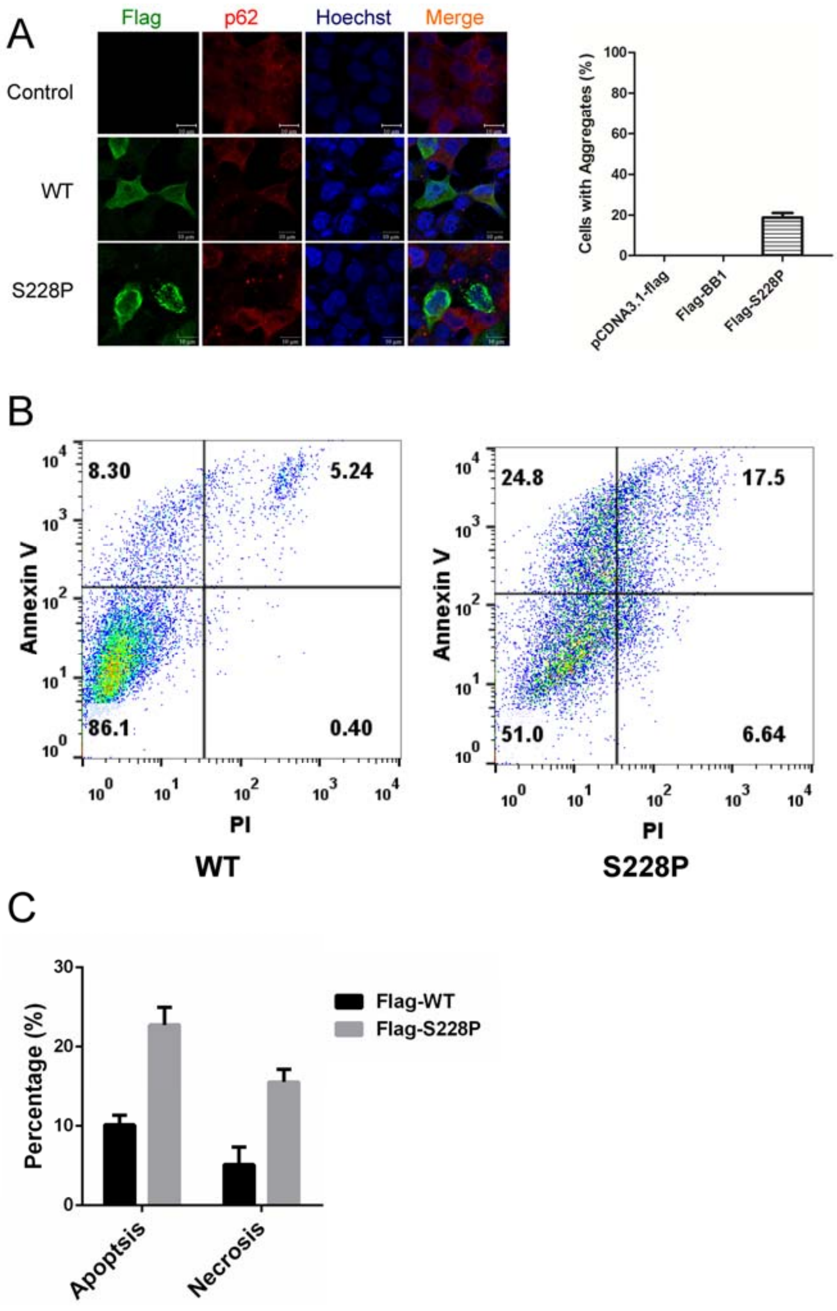

Supplementary Figure S3. The S228P mutation of βB1 induces cell death. (A) Proteins tagged by Flag at the N-terminus had similar intracellular distributions to proteins tagged by GFP at the N-terminus (Figure 1). The left panel shows the representative confocal images, while the right panel shows the quantitative analysis of cells containing crystallin aggregates. (B) Representative flow cytometry profiles of cells double stained by annexin V and PI. (C) Quantitative analysis of the percentages of apoptotic and necrotic cells. The presented data were from three independent experiments.

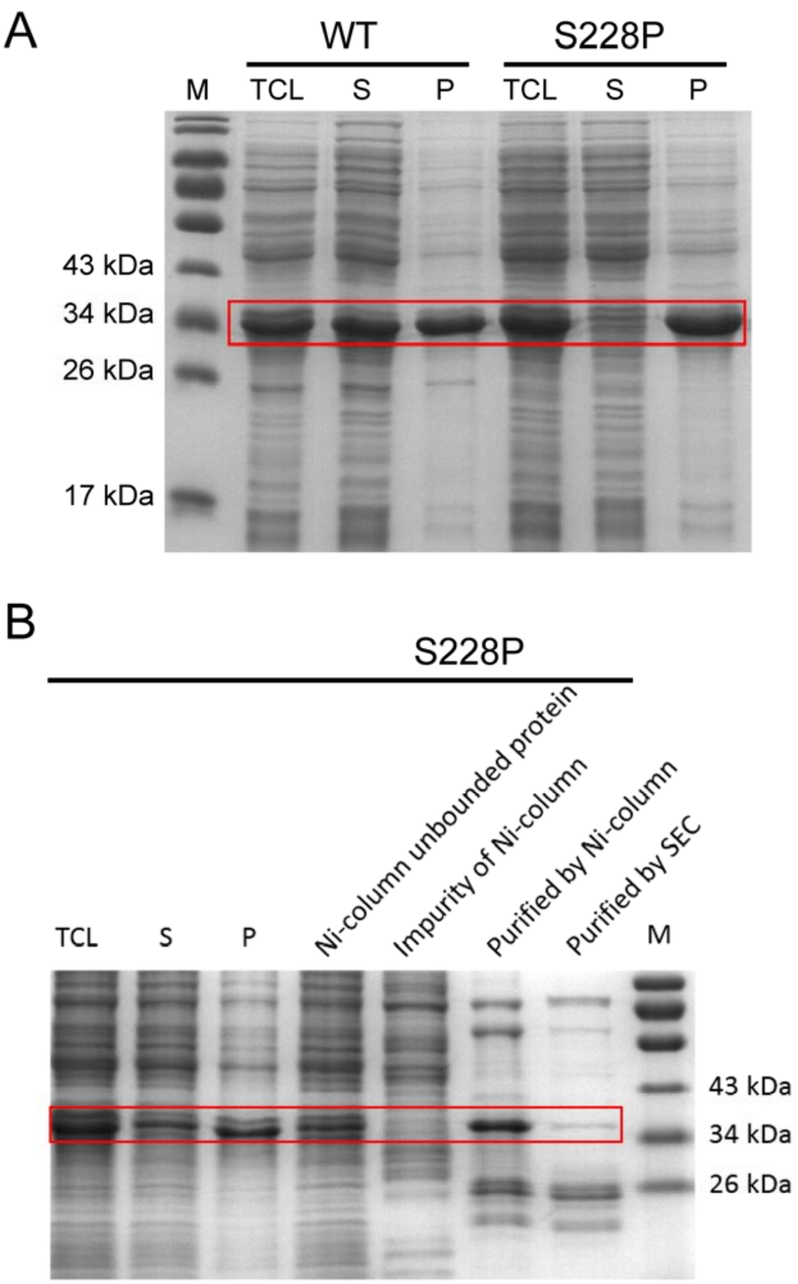

Supplementary Figure S4. The S228P mutation promotes inclusion body formation in *E. coli* cells. (A) SDS-PAGE analysis of overexpressed WT and mutated βB1 in *E. coli* cells cultured at 37°C. (B) SDS-PAGE analysis of overexpressed S228P in *E. coli* cells cultured at 12°C. TCL, total cell lysates; S, supernatant; P, precipitation; M, marker. Positions of the target proteins are highlighted by red box.

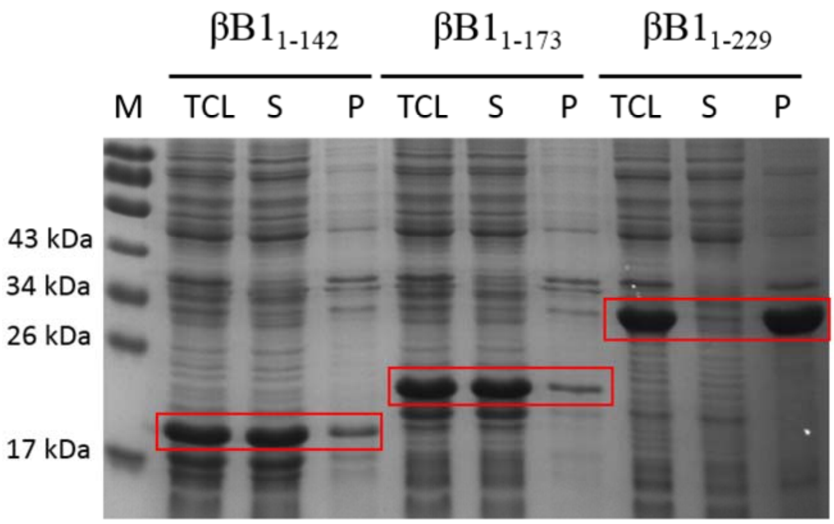

Supplementary Figure S5. SDS-PAGE analysis of overexpressed βB1 N-terminal fragments in *E. coli* cells. Positions of the target proteins are highlighted by red box. TCL, total cell lysates; S, supernatant; P, precipitation; M, marker.

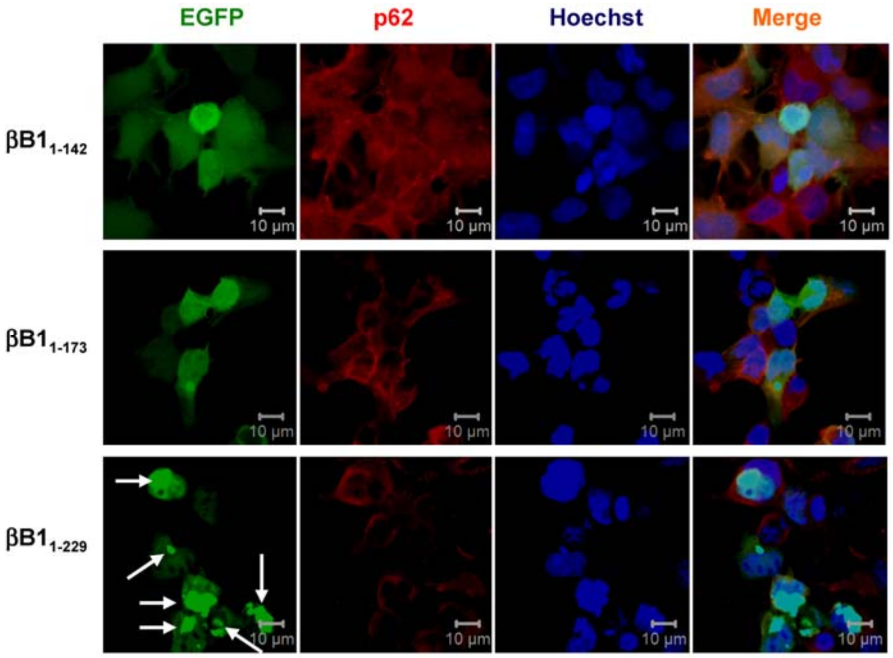

Supplementary Figure S6. Representative confocal images of βB1 proteolytic fragments expressed in the HEK 293T cells. The recombinant plasmids were constructed using pEGFP-C3. The truncated βB1 proteins were visualized by the tagged GFP (green). The aggresomes were recognized by the marker protein p62 (red). The nucleus was stained by Hoechst 33342 (blue). Positions of the aggregates are highlighted by white arrows.

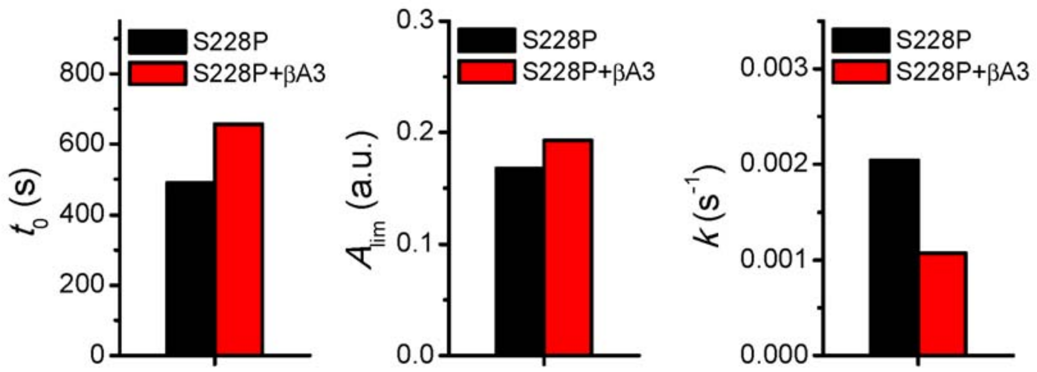

Supplementary Figure S7. Kinetic parameters of S228P aggregation in the presence and absence of βA3 at 37°C. The kinetic parameters were obtained by fitting the raw data shown in Figure 3G by the first-order aggregation kinetics. t_0, lag time; A_{lim}, turbidity (absorbance at 400 nm) at infinite time; k, aggregation rate.

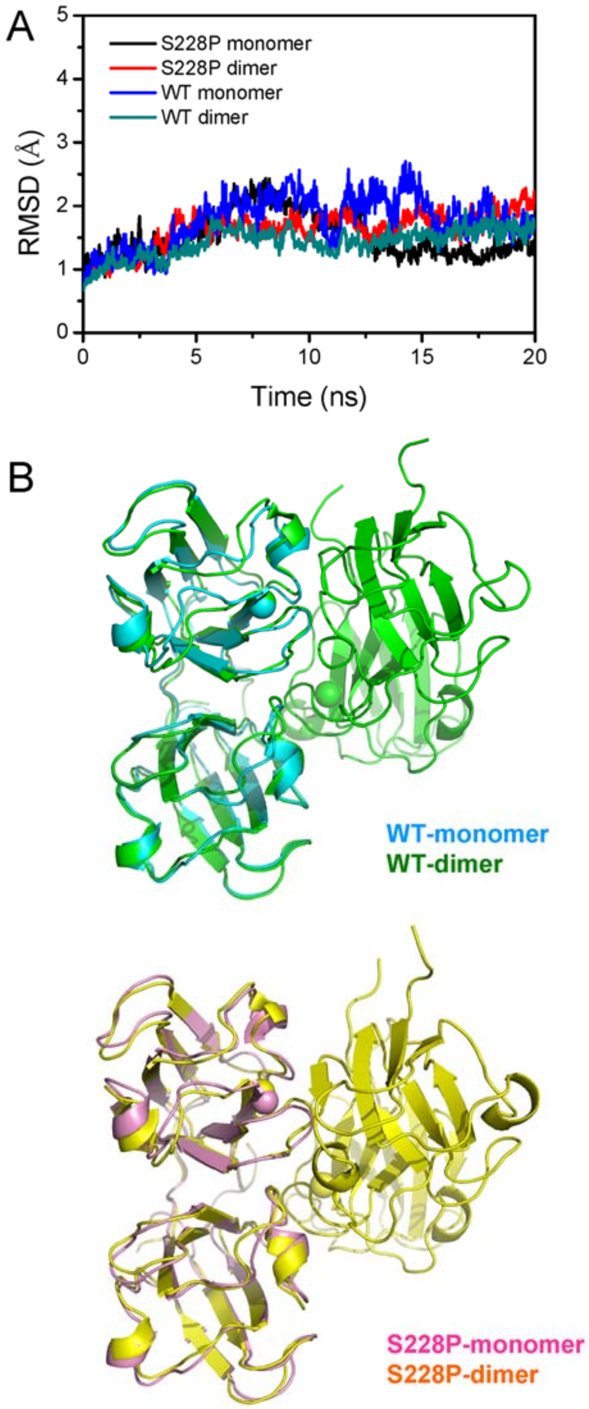

Supplementary Figure S8. Structural variations during MD simulations. (A) Time-course changes of the RMSD values during MD simulations. (B) A comparison of the last frame of the simulated dimeric and monomeric structures.

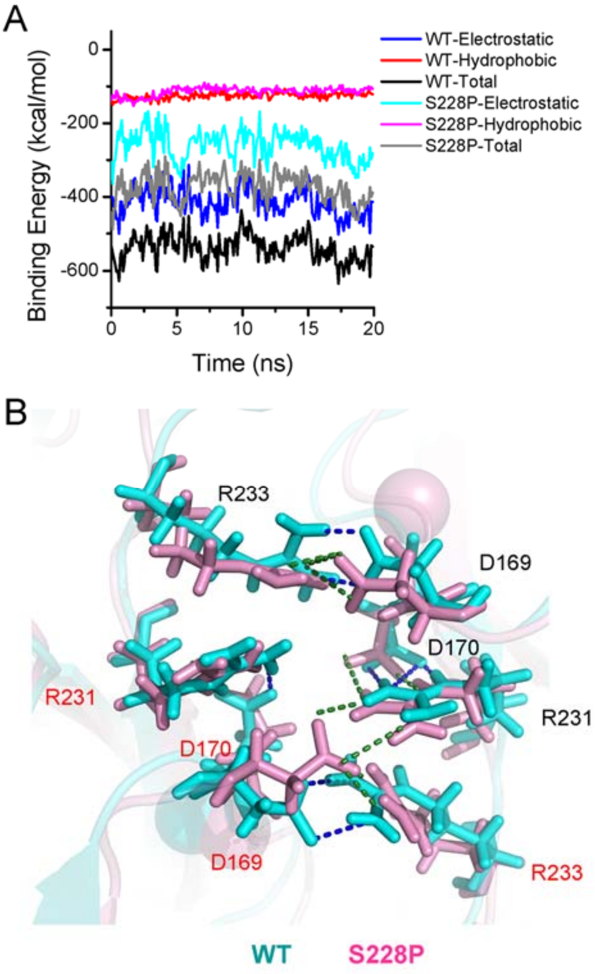

Supplementary Figure S9. Changes in subunit binding energy and interface structure caused by the S228P mutation. (A) Time-course change in subunit binding energy during MD simulations. (B) A comparison of the major H-bonding network forming the subunit interface. The positions of Cα atoms of residue 228 are shown in spheres.

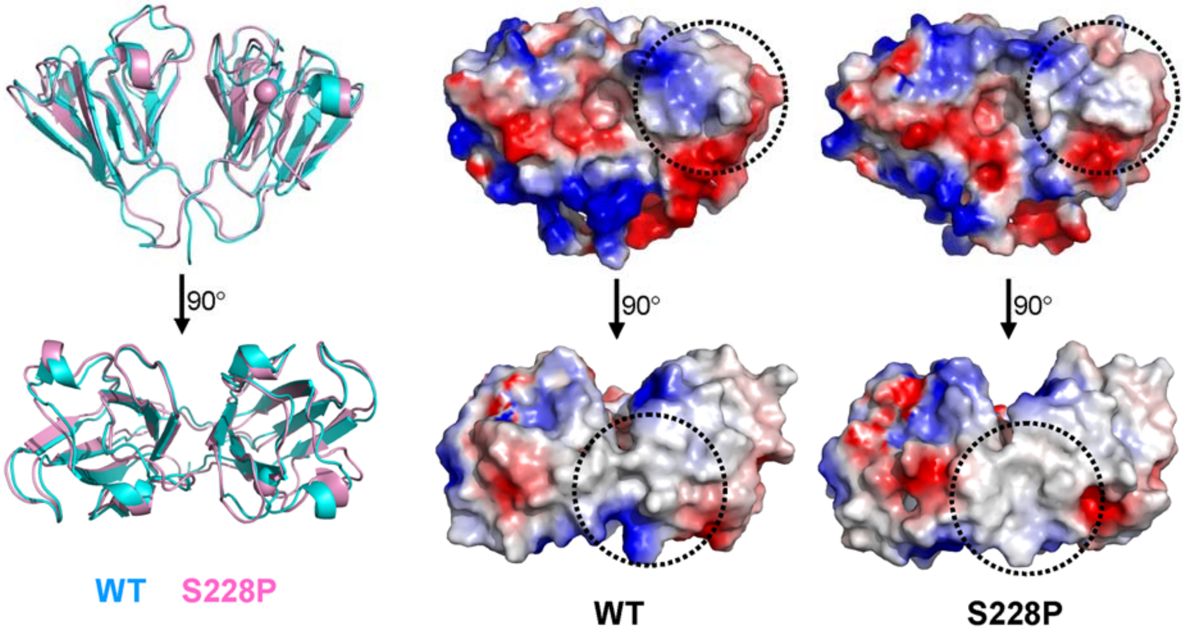

Supplementary Figure S10. A comparison of the simulated monomeric structures and surface electrostatic potentials of the WT and S228P mutated proteins. Typical areas with changes in surface electrostatic potentials are indicated by dotted circles.

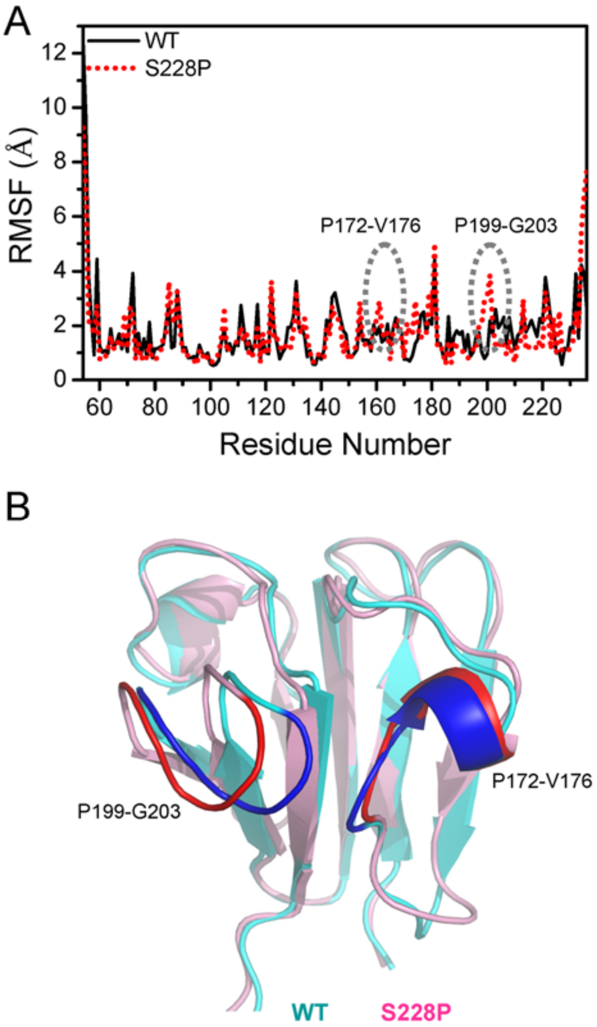

Supplementary Figure S11. Identification of regions with large structural fluctuations. (A) Time-course change in RMSF values during simulations. The circles show the regions that had large fluctuations in the mutant but not in the WT protein. (B) Positions of the regions with large structural fluctuations caused by the mutation. In the WT protein, the two regions form interacting loops to shield the hydrophobic core of the C-terminal domain from solvent access.
